# Four genetic loci control compact plant size with yellow pear‐shaped fruit in ornamental tomato (*Solanum lycopersicum* L.)

**DOI:** 10.1002/tpg2.20017

**Published:** 2020-04-11

**Authors:** Meysam Safaei, Jamal‐Ali Olfati, Yousef Hamidoghli, Babak Rabiei, Eiji Yamamoto, Kenta Shirasawa

**Affiliations:** ^1^ Department of Horticultural Sciences, Faculty of Agricultural Sciences University of Guilan Rasht Guilan Iran; ^2^ Department of Frontier Research and Development Kazusa DNA Research Institute Kisarazu Chiba 292‐0818 Japan; ^3^ Department of Agronomy and Plant Breeding, Faculty of Agricultural Sciences University of Guilan Rasht, Gilan Province Iran

## Abstract

Tomato is an attractive fruiting vegetable crop that can be used as an ornamental plant. Agronomical traits have been subjected to extensive genetic dissection to enhance vegetable breeding programs. By contrast, there are few genetic studies of ornamental traits for the development of ornamental tomato varieties. To investigate genetic loci linked to desired ornamental traits, we performed genetic analyses using an intraspecific mapping population that segregated for fruit color (yellow or red), fruit shape (round or pear), and plant height (high or compact). A genetic map was constructed with 965 single nucleotide polymorphisms (SNPs) and 33 simple sequence repeat markers. Subsequent linkage analysis using quantitative locus analysis and genome‐wide association study detected four genetic loci for the three selected traits, all of which were located near the reported genes. We performed KASP—kompetitive allele‐specific PCR—to develop SNP markers that were tightly linked to the four loci. Highly accurate genotyping data were obtained from the four SNPs across 187 F2 plants, which enabled us to select two lines with homozygous alleles for compact plant size and yellow pear‐shaped fruits. These newly developed SNP markers and genetic strategies could be used to accelerate breeding programs for ornamental tomato plants.

**Abbreviations**
ddRAD‐seq: double‐digest restriction site‐associated DNA sequencing
EST: expressed sequence tag
GWAS: genome‐wide association study
KASP: kompetitive allele‐specific PCR
LG: linkage group
LOD: logarithm of the odds
MAS: marker‐assisted selection
NGS: next‐generation sequencing
QTL: quantitative trait locus
SNPs: single nucleotide polymorphisms
SSR: simple sequence repeat

## 1 INTRODUCTION

Tomato (*Solanum lycopersicum* L.) is one of the most important vegetable crops cultivated globally. Tomato plants are also used as ornamentals, although the breeding programs for crop and ornamental varieties are substantially different. For example, ornamental plants require a compact size so that plants can thrive under potted conditions rather than in fields and greenhouses (Scott & Harbaugh, 1989, 1995; Scott, Harbaugh, & Baldwin, 2000). Fruit color variations are also attractive traits in ornamental plants. These traits are recognized as indeterminate qualitative traits that are primarily controlled by single genes exhibiting Mendelian inheritance. Therefore, a gene‐pyramiding strategy would be a promising and efficient approach for developing attractive ornamental cultivars (Pedersen & Leath, 1988). Marker‐assisted selection (MAS) can enhance the efficiency of gene‐pyramiding strategies (Servin, Martin, Mezard, & Hospital, 2004).

Many genes expressing quantitative and qualitative traits have been reported in tomato (Tomato Genome Consortium, 2012). Some genes could be useful for breeding programs for ornamental tomato cultivars. Multiple gene loci are sometimes linked with a single trait (Rodriguez et al., 2011). For example, tomato plant height is determined by mutations in *DWARF* (Bishop, Harrison, & Jones, 1996) and *SELP‐PRUNING* (Pnueli et al., 1998) genes. Fruit color is governed by *Y* (Ballester et al., 2010) and *R* (Bartley et al., 1992) genes, which control the colors of the epidermis and flesh, respectively. There are several candidate genes governing fruit shape, including *OVATE* (Liu, Van Eck, Cong, & Tanksley, 2002), *LOCULE NUMBER* (Munos et al., 2011), *FASCIATED* (Cong, Barrero, & Tanksley, 2008), and *SUN* (Xiao, Jiang, Schaffner, Stockinger, & van der Knaap, 2008). Most of these genes have been identified by gene mapping populations derived from interspecific crosses because of the large number of polymorphisms over the genome (Shirasawa & Hirakawa, 2013). However, it would be efficient to use intraspecific crosses (cultivated × cultivated lines) because they seldom require repeated backcrossing to eliminate linkage drags from wild relatives (Zamir, 2001). To perform gene pyramiding using a specific combination of cultivated lines, it is necessary to know which genetic loci are segregated in the breeding populations.

Genetic mapping is one of the most reliable methods to identify genetic loci (or genomic regions) that control traits in the populations. However, low polymorphism rates in intraspecific populations are technically challenging during genetic mapping (Shirasawa & Hirakawa, 2013). Therefore, it is necessary to identify a sufficient number of polymorphic markers to develop genetic maps using traditional PCR‐based markers such as simple sequence repeat (SSR) or microsatellite markers (Shirasawa et al., 2010b). High‐throughput genotyping methods based on next‐generation sequencing (NGS) technology can overcome some of these technical challenges (Davey et al., 2011). Restriction site‐associated DNA sequencing (RAD‐seq) (Baird et al., 2008) and genotyping‐by‐sequencing (Elshire et al., 2011) provide genome‐wide polymorphic data, including single nucleotide polymorphisms (SNPs). Our previous study showed that double‐digest RAD‐seq (ddRAD‐seq) (Peterson, Weber, Kay, Fisher, & Hoekstra, 2012), an alternative technique of RAD‐seq, effectively generates genome‐wide SNPs in an intraspecific population of tomato (Shirasawa, Hirakawa, & Isobe, 2016).

It is necessary to convert the NGS‐based SNPs into diagnostic markers that can be used in breeding programs with MAS (Arafa & Shirasawa, 2018). Kompetitive allele‐specific PCR (KASP) is a popular SNP genotyping technology due to low cost and high reliability (He, Holme, & Anthony, 2014). In this study, we pyramid three phenotypes (compact plant size, yellow‐colored fruit, and pear‐shaped fruit) in cultivated tomato and genetically map the three traits by performing SSR and ddRAD‐seq analyses. Polymorphisms linked to the traits were converted into KASP markers for breeding programs based on MAS. We compared the genotyping accuracies of ddRAD‐seq and KASP techniques. These strategies and results can be used to develop effective SNP markers for MAS‐based breeding programs.

**Core Ideas**

- Tomato can be used as an ornamental plant and a vegetable crop.
- Linkage mapping identified genetic loci for the ornamental traits.
- SNP markers tightly linked to the loci were developed to select plants of interest.
- This approach can accelerate breeding programs for ornamental tomatoes.

## 2 MATERIALS AND METHODS

### 2.1 Plant materials

We used two tomato inbred lines, P110 and M110, stocked in University of Guilan, Iran. P110 is an indeterminate growth type with yellow pear‐shaped fruits, whereas M110 has a miniature growth habit with round‐shaped red fruits. Two F2 mapping populations were generated from a reciprocal cross between the two lines. One was a forward F2 population (*n* = 92) derived from a cross between P110 (paternal) and M110 (maternal). The other reverse F2 population (*n* = 95) was derived from a cross between P110 (maternal) and M110 (paternal). High‐quality genomic DNA was extracted from leaves at early growth stages using the DNeasy Plant Mini kit (Qiagen, Hilden, Germany), and DNA quality and quantity were measured using the Lunatic UV/Vis reader (Unchained Labs, Pleasanton, CA, USA).

Seeds of the parental lines and the F2 populations were sown in a mixture of cocopit and perlite in a greenhouse, and were allowed to grow for one month before being transplanted into a field. Fruit shape was determined using the parameter “Height Mid‐width” implemented in Tomato Analyzer (Brewer et al., 2006), and fruit color was measured using MATLAB R2018b software (MathWorks, Inc., Natick, MA, USA). Plant height (cm) was measured during the growing period at five months after transplantation into the field.

### 2.2 SSR marker analysis

The genetic map tomato‐EXPEN 2000 (Shirasawa et al., 2010b) was used to select a total of 810 SSR markers for polymorphism screening between the two parental lines, including 307 genome SSRs (TGS) and 503 expressed sequence tag (EST)‐SSRs (TES) (Supplemental Table S1). Primer information for the tested markers is available at the Kazusa Marker Database (http://marker.kazusa.or.jp) (Shirasawa, Isobe, Tabata, & Hirakawa, 2014). The PCR and electrophoresis analysis using 10% polyacrylamide gels were performed as described previously (Shirasawa et al., 2010b).

### 2.3 ddRAD‐seq analysis

A ddRAD‐seq library was constructed as described previously (Shirasawa et al., 2016) with minor modification. Briefly, genomic DNA from parental and forward F2 populations was digested with two restriction enzymes, *Pst*I and *Msp*I (Fast Digest restriction enzymes; Thermo Fisher Scientific, Waltham, MA, USA), and ligated with index adapters (Supplemental Table S2). Nucleotide sequences were determined using NextSeq500 (Illumina) in paired end, 76 bp mode. Data processing and SNP identification were performed as described previously (Shirasawa et al., 2016). High‐quality sequence reads were mapped to the reference sequence of the tomato genome SL3.0 (Shearer et al., 2014) to detect high‐confidence SNPs.

### 2.4 Genetic map construction, quantitative trait loci (QTL) analysis, and genome‐wide association study (GWAS)

Segregating data of SSR and SNP loci were used for linkage analysis using Joinmap version 4 (Van Ooijen, 2006). Marker loci showing similarity greater than 0.95 were removed. Linkage groups were first defined using a logarithm of the odds (LOD) threshold of 2–10. The Kosambi function was used to translate the recombinant ratios into map distances in centimorgan (cM). The resulting map was drawn with MapChart (Voorrips, 2002).

QTL were detected for each of the traits using the multiple QTL mapping method implemented in MAPQTL version 5 (Van Ooijen, 2004). LOD significance thresholds (*p* < .05) were analyzed by running 1,000 permutation tests. Association mapping for quantitative traits was performed using the general linear model implemented in TASSEL version 5.0 (Bradbury et al., 2007). Association mapping of fruit color as a binary trait was performed using the glm function in R version 3.6.1 (https://www.r‐project.org) with the argument family equal to binomial. Statistical significance was evaluated using the chi‐square test of differences in deviance between the null model and the model with SNP genotype. The thresholds for the association were set as 5.2 × 10^−5^ (= 0.05/965) at a significance level of 5% after Bonferroni multiple test correction.

### 2.5 KASP assay

KASP markers were designed for SNPs linked to traits detected in QTL analysis and GWAS. The KASP assay was performed according to the manufacturer's protocol (http://lgcgenomics.com). Fluorescent signals were detected using a 7900HT Fast Real‐Time PCR System (Applied Biosystems), and data were analyzed with SDS version 2.3 (Applied Biosystems).

## 3 RESULTS

### 3.1 Distribution of phenotypic variations

**FIGURE 1:**
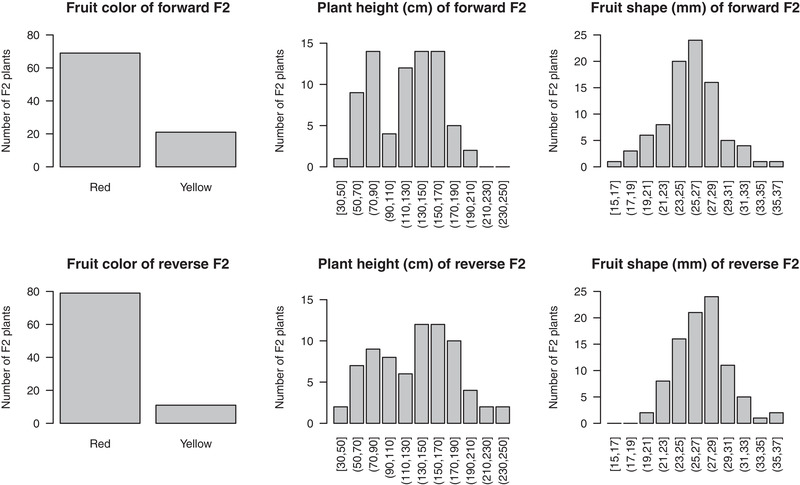
Phenotype distributions in the forward and reverse F2 populations

The phenotypes of three traits differentially segregated in the two F2 populations (Figure 1; Supplemental Table S3). Of the 187 lines (92 forward + 95 reverse) across the two F2 populations, fruit phenotypes were available for 180 plants (90 forward + 90 reverse) while the remaining seven had no fruit at the time of fruit phenotyping. In addition, plant height data were available for 149 lines (75 forward + 74 reverse), of which 38 died before plant height measurement at five months after transplantation into the field.

Fruit color exhibited binomial distribution (yellow or red) in the F2 population. The numbers of forward F2 lines with yellow and red fruits were 21 and 69, respectively. This pattern fit the Mendelian segregation ratio for a single gene (*p *= 0.715, χ^2^ = 0.133), and suggested that the red fruit phenotype was dominant over yellow fruit, which is supported by red fruit on the F1 plant. However, in the reverse F2 population, the numbers of lines with yellow and red fruits were 11 and 79, respectively, and their ratio was significantly different from that predicted by the single locus model (*p* = .005, χ^2^ = 7.837).

By contrast, the segregation patterns of plant height (ranging from 40 cm to 245 cm) and fruit shape (ranging from 15.5 mm to 36.1 mm) in F2 plants exhibited continuous distributions. These distribution patterns indicated that fruit shape (normal distribution pattern) and plant height (binomial distribution pattern) were polygenic and oligogenic inheritances, respectively.

### 3.2 Screening analysis of SSR markers

**FIGURE 2:**
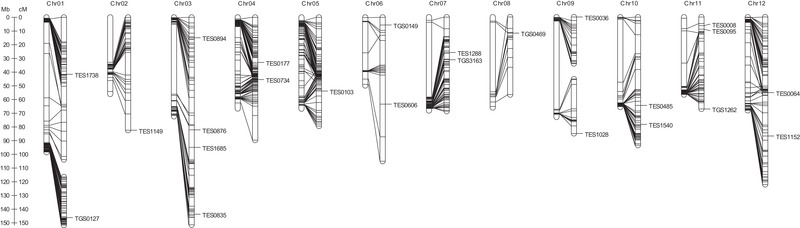
Maps of the F2 population derived from a cross between P110 and M110 *Note*: Physical maps of the tomato genome and linkage group maps based on SNPs are shown on the left side and right side, respectively. SSR markers with their names are indicated. Loci that are identical between the two maps are connected by lines.

A total of 810 SSR markers (503 TES and 307 TGS) are listed in Supplemental Table S1. Of these, 33 SSRs (25 TES and 8 TGS) displayed polymorphisms between the parental lines. The polymorphism rate of the SSR markers in this study was as low as 4.1% (5.0% in TES and 2.6% in TGS). The numbers of polymorphic markers on each chromosome ranged from 1 (chromosomes 5 and 8) to 4 (chromosomes 3, 10, 11, and 12) (Figure 2; Table 1; Supplemental Table S3). Segregating data were obtained from all 33 SSR markers across the forward F2 mapping population.

**TABLE 1:** Genetic map of the F2 population derived from a cross between P110 and M110

Linkage group	Map length (cM)	No. of SNPs	No. of SSRs
1.1	103.8	38	1
1.2	35.2	51	1
2	82.4	33	1
3	151.4	58	4
4	89.6	90	2
5	79.4	76	1
6	105.0	17	2
7	68.7	56	2
8	56.7	11	1
9.1	34.0	18	1
9.2	39.7	12	1
10	93.5	43	2
11	67.1	39	3
12	122.2	67	2
Total	1128.7	609	24

### 3.3 ddRAD‐seq analysis

To identify additional genetic markers, we performed ddRAD‐seq analyses for the two parental lines, an F1 line, and a forward F2 line (*n* = 92). We obtained a total of 5.1 Gb of raw data, which represents a mean of 53.5 Mb for each line (Supplemental Table S2). The sequence data were mapped with an alignment rate of 97.8% on the tomato genome sequence (SL3.0), as a reference to detect 965 SNPs. The numbers of SNPs varied from 14 on chromosome 8 to 153 on chromosome 1 (Figure 2; Table 1; Supplemental Table S4).

### 3.4 Construction of a genetic map

A total of 998 polymorphic markers (33 SSR markers and 965 SNPs) were used for linkage analysis. Of these, 633 loci were classified into 14 linkage groups (LGs) corresponding to tomato chromosomes, except for chromosomes 1 and 9 with two groups (Figure 2; Table 1; Supplemental Table S4). The total map length was 1,128.7 cM, with an average length of 2.5 cM between two adjacent loci. The numbers of markers on the linkage groups ranged from 12 on LG8 to 92 on LG4.

### 3.5 Linkage analysis with QTL analysis and GWAS

**FIGURE 3:**
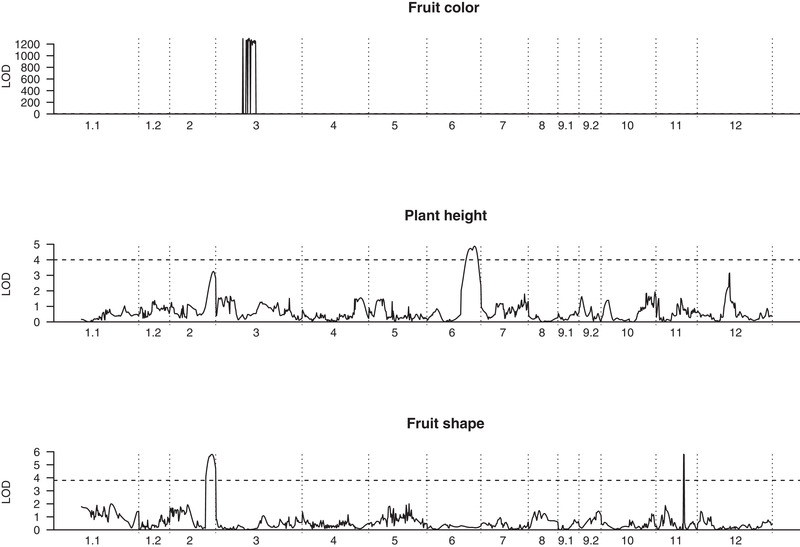
Map positions of QTLs for fruit color, plant height, and fruit shape *Note*: Vertical dash lines are threshold values for LOD scores determined from 1000 permutation tests. The peaks on the LOD curves above the threshold lines indicate the putative positions of the QTLs.

The QTL analysis determined the threshold values for LOD scores using 1,000 permutation tests for each trait. Four QTLs were detected for the three traits: one on LG3 for fruit color; two on LG2 and LG11 for fruit shape; and one on LG6 for plant height (Table 2; Figure 3). In parallel, we performed a GWAS analysis using 965 SNPs. The GWAS analysis detected one significant peak (at 4,462,072 bp on chromosome 3) for fruit color and two significant peaks (at 54,104,795 on chromosome 2 and 54,879,959 on chromosome 11) for fruit shape (Supplemental Figure S1). All of these peaks corresponded to QTL positions. We detected a total of four genetic loci for the three traits. The genome positions of these loci were close to the reported genes for the traits: *R* (Solyc03g031860) for fruit color; *SELF‐PRUNING* (Solyc06g074350) for plant height; and *OVATE* (Solyc02g085500) and *FASCIATED* (Solyc11g071810) for plant shape (Table 2).

**TABLE 2:** QTLs for fruit color, plant height, and fruit shape

Trait	Linkage group	Position (cM)	Marker interval	LOD of peak	Phenotypic variation explained (%)	Additive effect^a^	Dominant effect^a^	Candidate gene	Gene ID of the candidate
Color	3	55.4	SL3.0ch03_4462072 – SL3.0ch03_5059867	1298.1	17.9	−0.5	0.5	*R*	Solyc03g031860
Height	6	92.2	SL3.0ch06_45138197 – SL3.0ch06_48884281	4.9	35.0	29.5	23.8	*SELF‐PRUNING*	Solyc06g074350
Shape	2	74.4	SL3.0ch02_46156826 – TES1149	5.8	21.6	2.5	0.2	*OVATE*	Solyc02g085500
Shape	11	47.2	SL3.0ch00_11482242 – SL3.0ch11_54879959	5.8	20.0	1.1	2.9	*FASCIATED*	Solyc11g071810

a Effect of P110 alleles.

### 3.6 KASP assay to validate the QTLs

The linkage analysis results were validated by SNP genotyping across the two mapping populations, forward and reverse F2s. One, one, and two SNPs for fruit color (SL3.0ch03_4462072), plant height (SL3.0ch06_45138197), and fruit shape (SL3.0ch06_45138197 and SL3.0ch02_54104795), respectively, were selected at the candidate regions to design the KASP assay kits.

**FIGURE 4:**
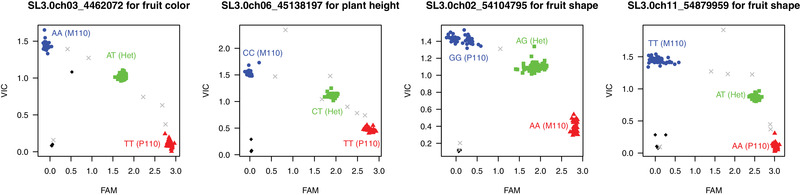
Signal clusters of the KASP assays for SNPs linked to the four target traits *Note*: *x* and *y* axes are fluorescent signal intensities of FAM and VIC, respectively. Genotypes of homozygotes of VIC, heterozygotes, and homozygotes of FAM are represented by circles, squires, and triangles, respectively. Negative controls are indicated by rhombuses and unknowns are indicated by crosses.

KASP analysis resulted in 358 genotyping scores in the forward F2 population (*n* = 92) (Figure 4), whereas the remaining scores were missing. These data were compared with the genotype calls from ddRAD‐seq analysis. A total of 349 data points were commonly genotyped in KASP and ddRAD‐seq, with 345 identical data points and four unique points. The mismatched SNPs included two for chromosomes 2 and 3 in fF2‐00638, and two for chromosomes 3 and 11 in fF2‐21624. We did not find any clear reasons for the mismatches. The plots of the four unique data points were positioned in each fluorescent signal cluster (Supplemental Figure S2), and the sequence alignments of the ddRAD‐seq reads at the four SNP sites were not disrupted (Supplemental Figure S2). The concentrations and qualities (A_260_/A_280_ and A_260_/A_230_, respectively) of genomic DNA extracted from the two samples were within the distributions of all tested samples (Supplemental Figure S3). We subsequently investigated the reproducibility of KASP assays using a kit for the SNP on chromosome 11 that was redesigned to analyze the forward F2 plant genotypes. All samples except one (fF2‐00638) had identical genotypes to those observed in the first KASP result (Supplemental Figure S4).

In the reverse F2 population (n = 95), KASP analysis resulted in 369 genotyping scores with 11 missing data. In accordance with the genotyping data, we selected two plants (fF2‐00654 and rF2‐02256) as homozygotic lines for the desired alleles (compact plant height, yellow fruit color, and pear‐shaped fruit) at the four QTLs. As shown in Supplemental Table S3, the fruit phenotypes of fF2‐00654 and rF2‐02256 were a yellow color and heights of 23.2 mm and 28.5 mm, respectively. rF2‐02256 was compact with a plant height of 58 cm but the plant height of fF2‐00654 was unavailable.

## 4 DISCUSSION

We identified genetic loci controlling plant height, fruit color, and fruit shape in the cultivated tomato lines used in this study. Among the three traits, fruit color was a qualitative trait that showed red/yellow segregation. While the forward F2 population exhibited a 3:1 Mendelian ratio for red/yellow segregation, the reverse population exhibited a ratio significantly different from 3:1 (Figure 1). It is possible that cytoplasmic variations in these plants control color. However, the KASP results showed that fruit color was strongly associated with the SNP genotypes of SL3.0ch03_4462072 only. This might have been due to segregation distortion at this locus of the reverse F2 population. Thus, we concluded that DNA markers linked to these loci could be used for MAS in breeding programs to develop compact ornamental tomato lines with pear‐shaped yellow fruits.

We tested two polymorphism analysis techniques and confirmed that they have significantly different marker development efficiencies. The ddRAD‐seq technique is based on NGS technology and detected 965 SNPs across the F2 populations (*n* = 92) in one experiment. The numbers of detected SNPs were comparable with those detected between another combination of cultivated lines in our previous study (1,317 SNPs) (Shirasawa et al., 2016). This suggested that the ddRAD‐seq technique potentially detects approximately 1,000 SNPs between cultivated lines. By contrast, SSR analysis of 806 marker polymorphisms between parental lines only detected 33 markers (4.1%). This rate is as low as the percentage of polymorphic SSRs in intraspecies crosses (5.7%) (Shirasawa et al., 2010a) and remarkably less than that in interspecific combinations (28.1%) (Shirasawa et al., 2010b), although polymorphic rate depends on genetic distance between parental lines. We constructed a genetic map with the detected SSRs and SNPs. However, there were 14 LGs, more than the 12 tomato chromosomes. We assumed that some of the genome regions that were not covered with the genetic map had quite similar sequences in the two parental lines (Figure 2). Whole‐genome sequencing analysis could help to fill the gaps, whereas ddRAD‐seq detects only <1% of SNPs over the genome (Shirasawa et al., 2016).

The QTL analysis detected totals of one, two, and one genetic loci for fruit color, fruit shape, and plant height, respectively (Table 1; Figure 3). Based on the genome positions, these traits might be controlled by four well‐studied genes: *R*, *OVATE*, *FASCIATED*, and *SELF‐PRUNING*. Six genes have been reported to govern round‐shaped red or yellow fruits in ornamental tomato cultivars (Scott & Harbaugh, 1989, 1995; Scott et al., 2000). We proposed that among six genes, *OVATE* and *FASCIATED* might be required to produce pear‐shaped fruits in our populations at least and that the other genes might be monomorphic. Since only 633 loci (609 SNPs and 24 SSRs) of the 998 polymorphic markers (965 SNPs and 33 SSRs) were mapped on the 14 linkage groups employed in the QTL analysis, we performed GWAS with all 965 SNPs using the F2 population (Supplemental Figure S3). Three of the loci detected in the QTL analysis were significantly linked to the traits detected in GWAS, whereas the remaining locus for plant height was not detected. These results suggested that QTL analysis and GWAS have their own merits and limitations, and can be used to complement each other (Sonah, O'Donoughue, Cober, Rajcan, & Belzile, 2015). This might be one reason why interval mapping in QTL analysis enables estimates of genetic effects between each pair of adjacent markers. By contrast, GWAS can predict effects only at the marker positions. Linkage information to infer genotypes in the marker intervals could be used for mapping analysis, especially for qualitative traits.

We employed the KASP assay in MAS analysis to select plants with suitable traits for ornamental cultivars (He et al., 2014). We compared the genotyping scores for the four SNP loci across 92 samples of the forward F2 population. As 345 out of 349 genotyping scores were matched, the data accuracy was estimated to be 98.8%. The remaining four data points were mismatched; however, no potential reasons were found. We employed KASP to genotype 95 samples of the reverse F2 population, and identified two plant candidates out of 187 F2 plants (1.1%) for ornamental cultivars with yellow pear‐shaped fruits and compact plant height. Theoretically, due to the presence of homozygotic target alleles in all four target loci, one candidate line could be obtained from a population of 256 plants because this rate (1.1%) is close to the expected value of 0.4% (= 0.25^4^).

In conclusion, we identified four genetic loci to target in breeding programs for ornamental tomato cultivars. The results of this study suggest that our breeding strategy could be used to estimate the population size required to obtain desired plant traits.

## CONFLICT OF INTEREST STATEMENT

The authors declared that no competing interests exist.

## Supporting information

Supplemental Figure S1. Manhattan plots of GWAS for fruit color, plant height, and fruit shape.
Supplemental Figure S2. Comparison of genotyping scores in ddRAD‐Seq and KASP assay.
Supplemental Figure S3. Concentration and quality of genome DNA used for KASP assay.
Supplemental Figure S4. Comparison of two lots of KASP assay kit for the identical SNP.

Supplemental Table S1 Primer list of the SSR markers used in this study
Supplemental Table S2 Number of ddRAS‐Seq reads and rate of mapping against the reference genome sequence of tomato
Supplemental Table S3 Phenotypic data for the forward and reverse F2 populations
Supplemental Table S4 Marker positions on the genetic map of the F2 population derived from a cross between P110 and M110

## Data Availability

Sequence data are available at the DDBJ Sequence Read Archive database under the accession number DRA009249. SNP data from the ddRAD‐Seq analysis is available from KatomicsDB (http://www.kazusa.or.jp/tomato).
