# Supplementary material for: Four genetic loci control compact plant size with yellow pear‐shaped fruit in ornamental tomato (Solanum lycopersicum L.)
Source: Plant Genome. 2020 Apr 11;13(2):e20017. doi: 10.1002/tpg2.20017 (PMC12807392; doi:10.1002/tpg2.20017)
Supplement: Supplementary file 1 [file TPG2-13-e20017-s001.pdf]

Supporting Information

Four genetic loci control compact plant size with yellow pear-shaped fruit in ornamental tomato (*Solanum lycopersicum*)

Meysam Safaei[1,2], Jamal-Ali Olfati[1], Yousef Hamidoghli[1], Babak Rabiei[3], Eiji Yamamoto[2], and Kenta Shirasawa[2]*

[1]Department of Horticultural Sciences, Faculty of Agricultural Sciences, University of Guilan, Iran; [2]Department of Frontier Research and Development, Kazusa DNA Research Institute, Japan; and [3]Department of Agronomy and Plant Breeding, Faculty of Agricultural Sciences, University of Guilan, Iran

*** Corresponding author:** Kenta Shirasawa

Email: shirasaw@kazusa.or.jp

ORCID iD: 0000-0001-7880-6221

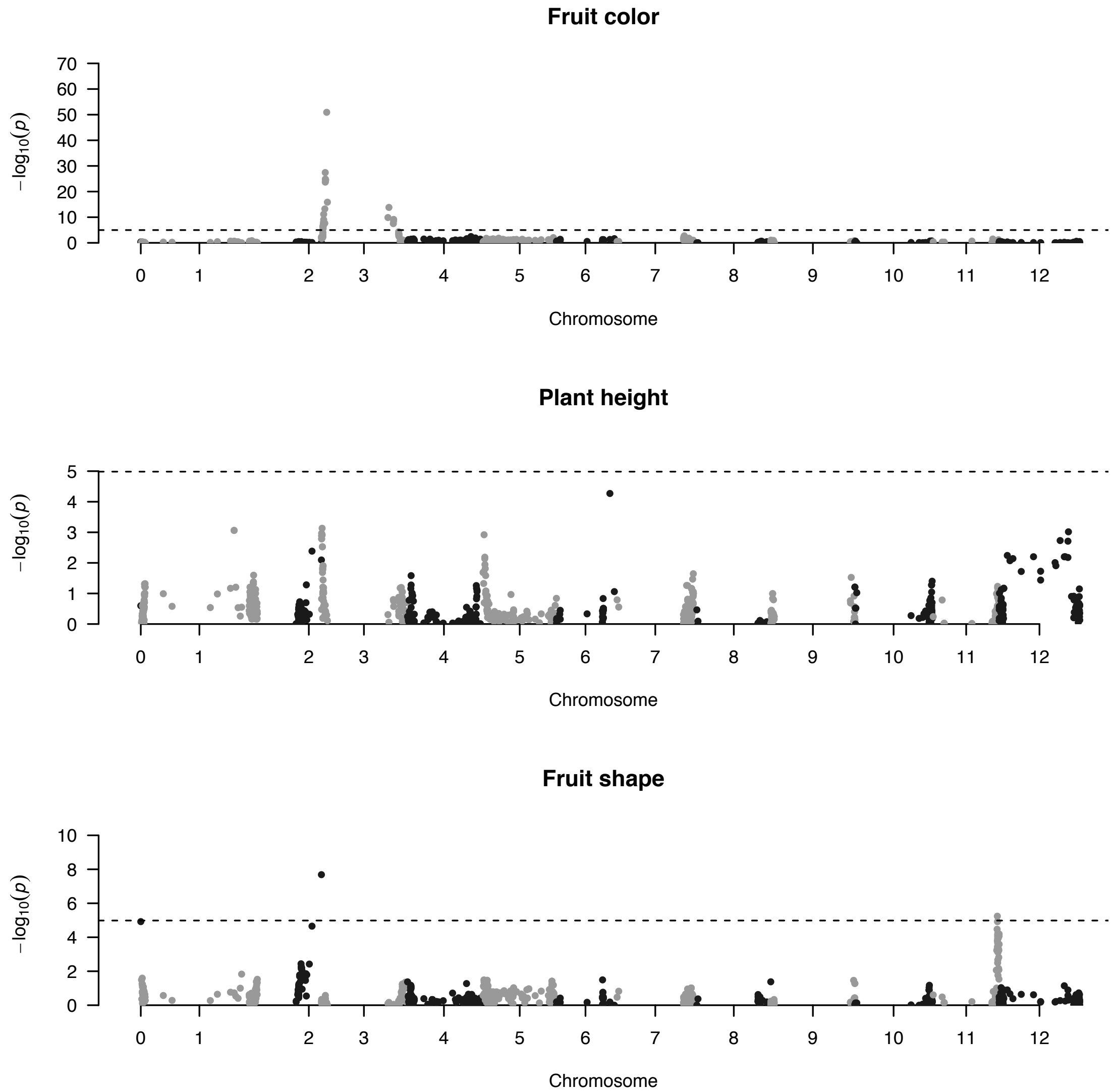

Supplementary Figure S1. Manhattan plots of GWAS for fruit color, plant height, and fruit shape.

Vertical dash lines are thresholds for the association at a significant level of 1% after Bonferroni multiple test correction. The peaks of the plots above the threshold lines indicate the putative position of the QTLs.

Shape (SL3.0ch02:54104795)

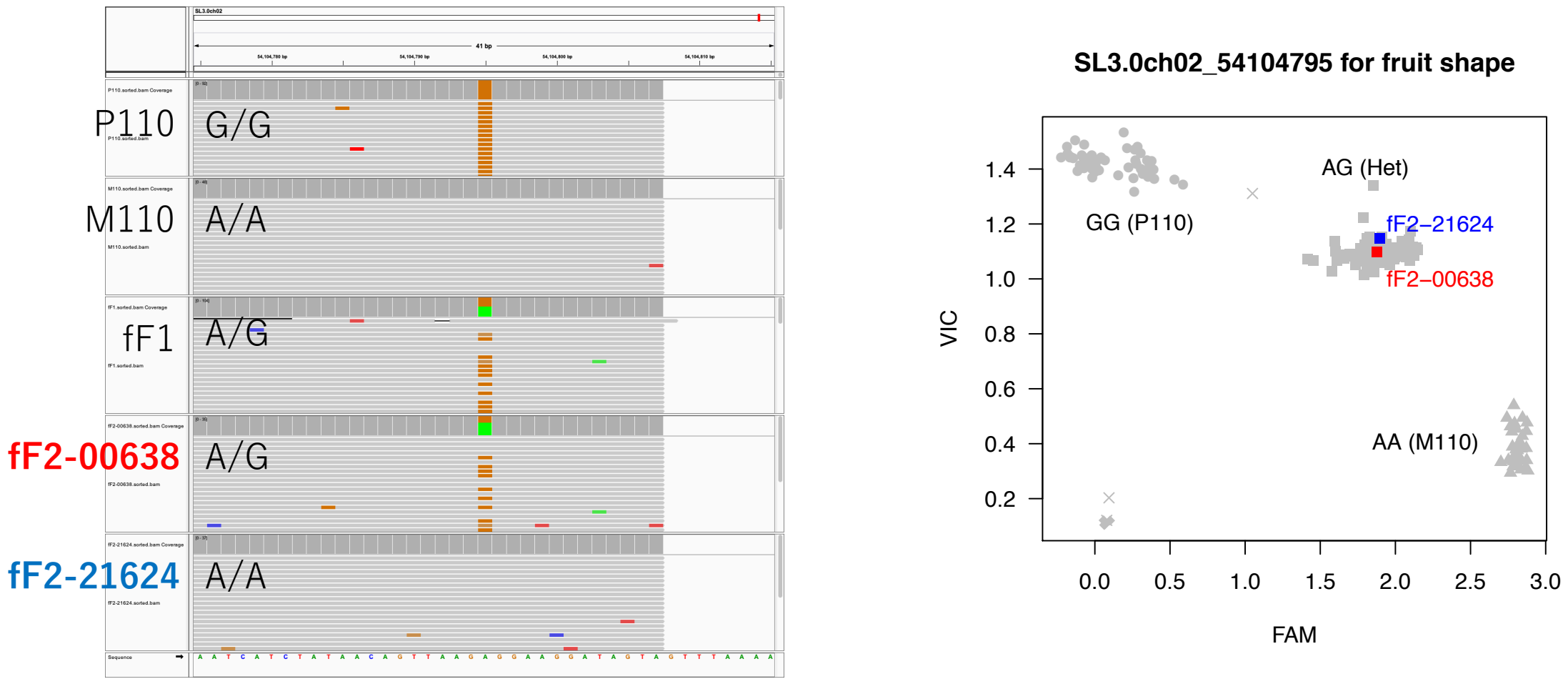

Color (SL3.0ch03:4462072)

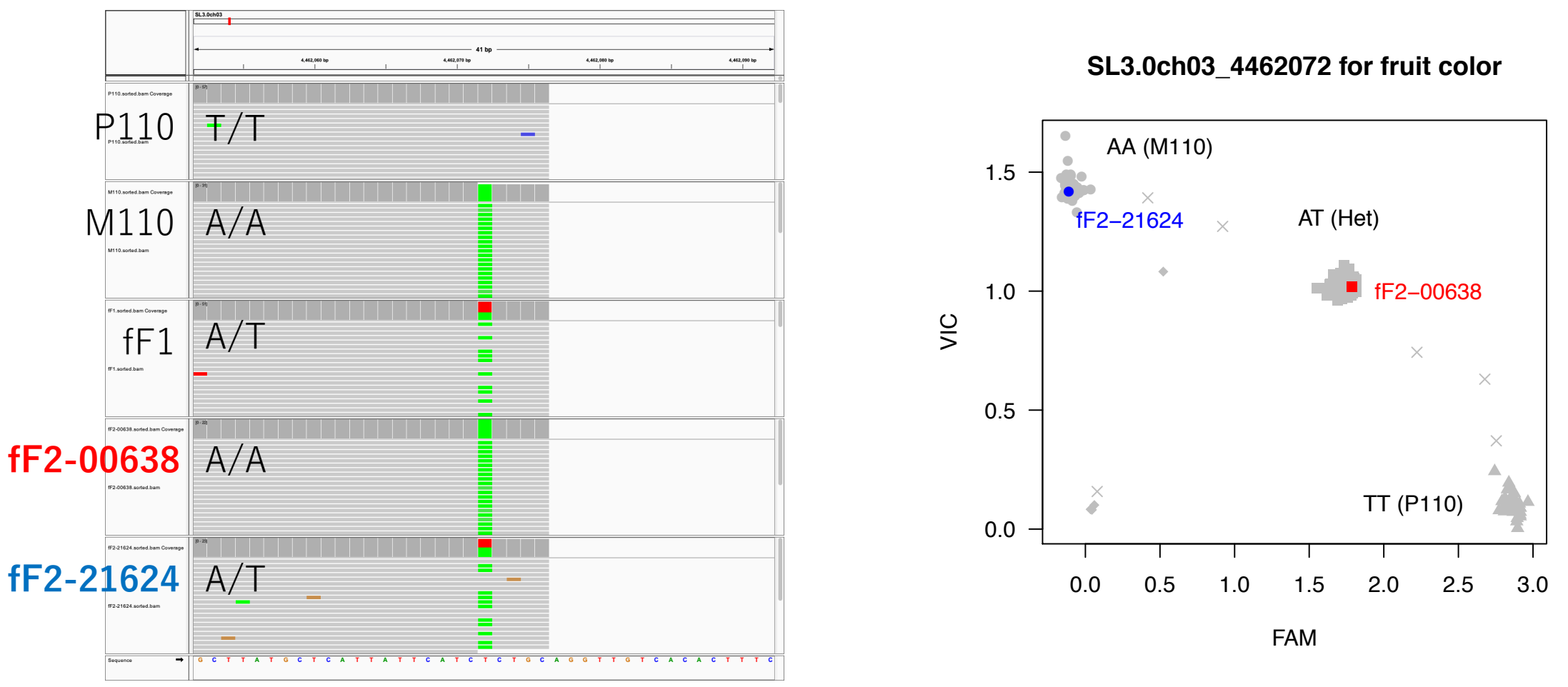

Shape (SL3.0ch11:54879959)

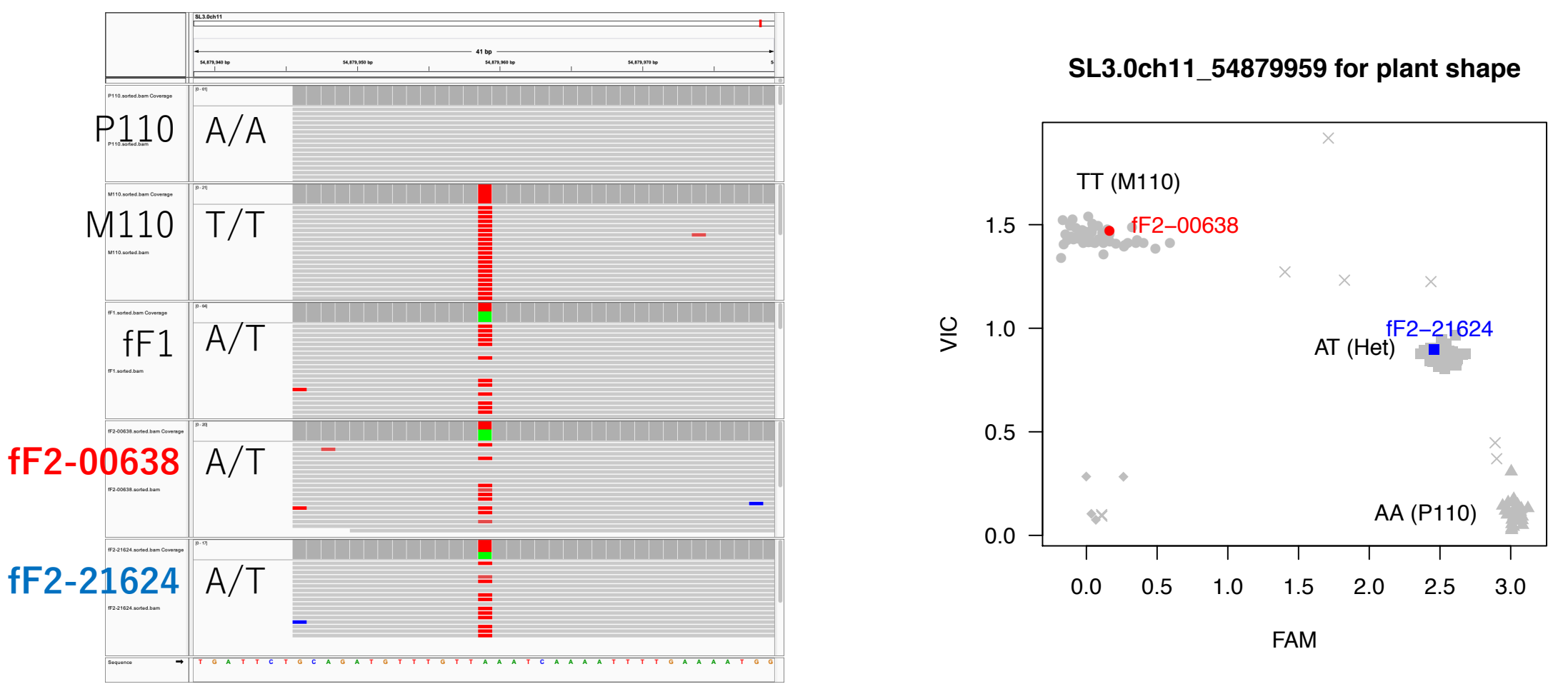

Supplementary Figure S2. Comparison of genotyping scores in ddRAD-Seq and KASP assay. The left panel shows sequence alignments of two F2 lines, fF2-00638 and fF2-21624, and their parental lines (P110, M110, and fF1) of the ddRAD-Seq reads. The right panel are signal

clusters of KASP assay. The two F2 lines, fF2-00638 and fF2-21624, are shown with red and blue plots. Genotypes of homozygotes of VIC, heterozygotes, and homozygotes of FAM are represented by circles, squires, and triangles, respectively. Negative controls are rhombuses and unknown are crosses.

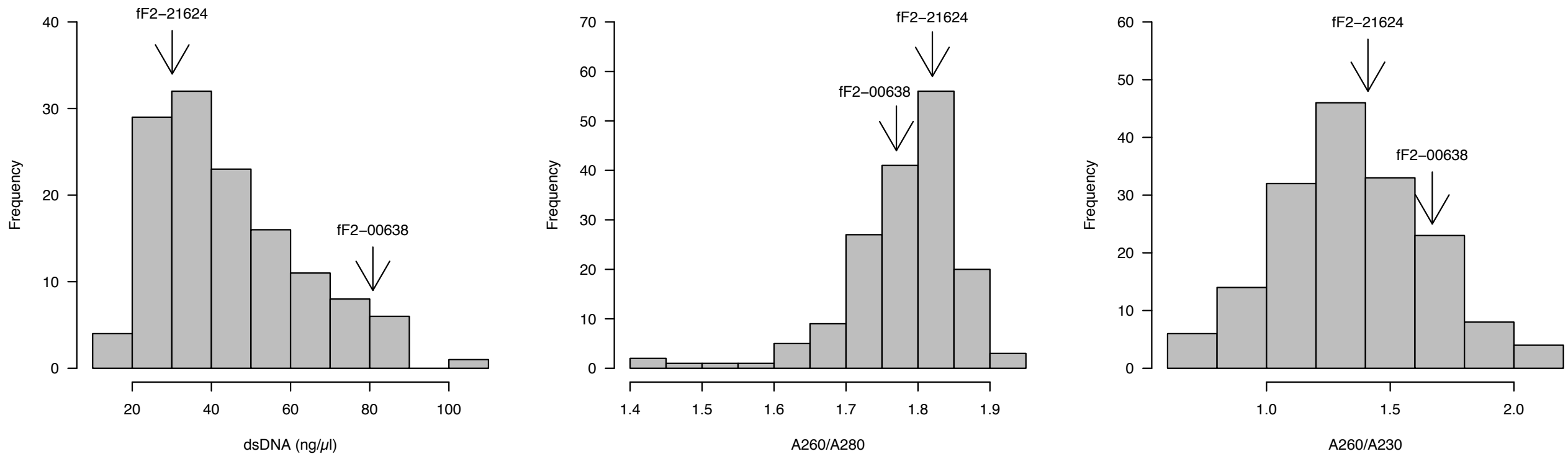

Supplementary Figure S3. Concentration and quality of genome DNA used for KASP assay. DNA quality (dsDNA, ng/µl) and quantity (A260/A280 and A260/A230) were measured using the Lunatic UV/Vis reader (Unchained Labs, Pleasanton, CA, USA).

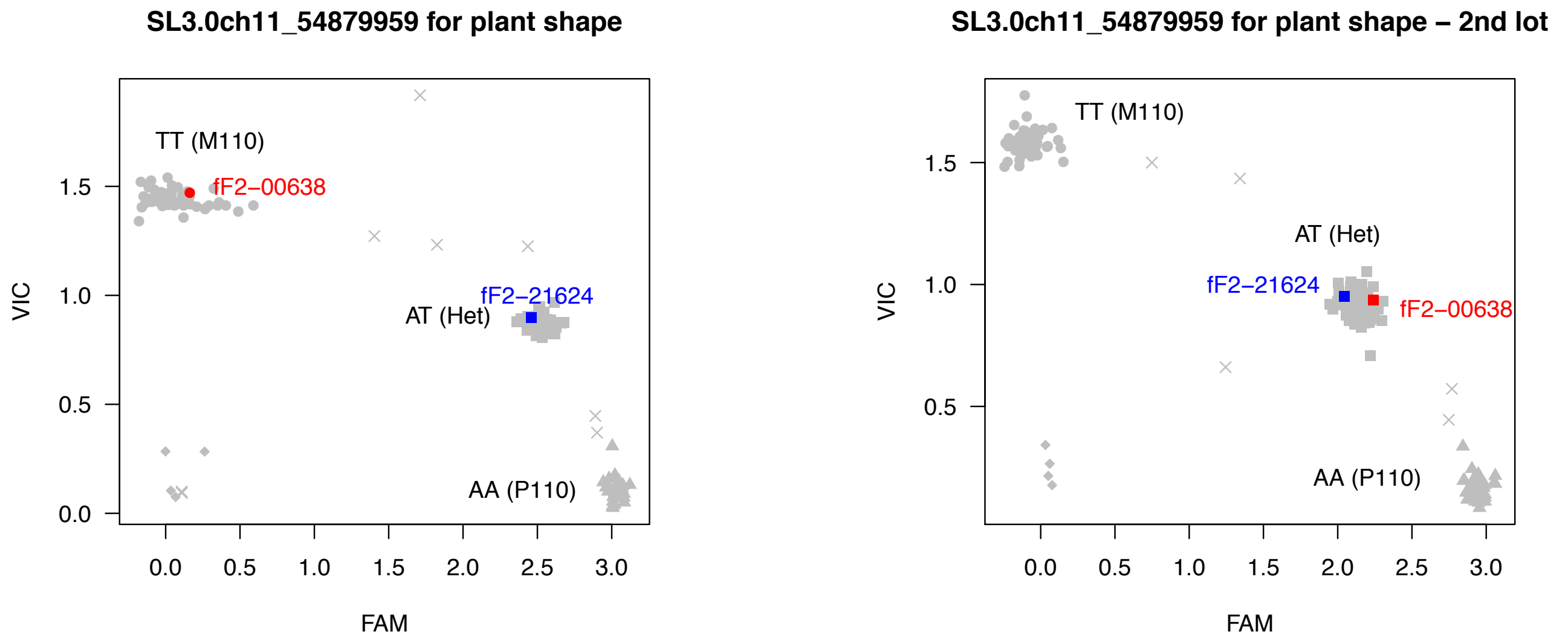

Supplementary Figure S4. Comparison of two lots of KASP assay kit for the identical SNP. The two F2 lines, fF2-00638 and fF2-21624, are shown with red and blue plots. Genotypes of homozygotes of VIC, heterozygotes, and homozygotes of FAM are represented by circles, squires, and triangles, respectively. Negative controls are rhombuses and unknown are crosses.
